# Chronic Constipation in the Elderly: An Unusual Presentation of Colonic Dysmotility in an Elderly Patient

**DOI:** 10.1155/2013/608505

**Published:** 2013-07-15

**Authors:** R. Peravali, H. Kranenburg, J. E. Martin, N. Keeling

**Affiliations:** ^1^Department of Colorectal Surgery, West Suffolk NHS Foundation Trust, UK; ^2^BICMS Pathology Group, Barts and The London School of Medicine and Dentistry, University of London, UK

## Abstract

*Introduction*. Chronic constipation is common in the elderly, and often no underlying pathology is found. Primary colonic dysmotility has been described in children but is rare in the elderly. * Case report.* We present an 82-year-old female with long standing constipation presenting acutely with large bowel obstruction. Laparotomy and Hartman's procedure was performed, and a grossly distended sigmoid colon was resected. Histology revealed a primary myopathic process. * Conclusion.* Primary colonic myopathy should be considered in elderly patients presenting with large bowel obstruction and a long preceding history of constipation, particularly when previous endoscopic examinations were normal.

## 1. Introduction

Primary colonic dysmotility secondary to a myapothic process has been described in children presenting with chronic constipation [1, 2]. Only two cases have been reported in adults aged 45 and 60 [3]. We report the only case in the English literature of primary colonic myopathy presenting acutely with large bowel obstruction in a patient over 80 years of age.

## 2. Case Report

This 82-year-old female presented with gross abdominal distension and lower abdominal pain as an emergency. Her normal bowel habit was that of longstanding constipation, but she had loose stools over the preceding week. Accompanied with this, she had a six-week history of nonspecific malaise, loss of appetite, and dizziness.

She had previously been admitted to a hospital 13 years ago with a 5-month history of abdominal distension, constipation, and faecal incontinence. Abdominal X-ray revealed gross faecal impaction which was managed successfully with phosphate enemas. Follow-up flexible sigmoidoscopy and biopsies revealed nonspecific colitis and melanosis coli.

Past medical history included hypertension and impaired glucose tolerance. Past surgical history was a previous caesarean section many years ago.

On examination, the patient was tachycardic (HR 115), hypotensive (BP 80/44), and tachypnoeic (RR 24). She had a nontender, grossly distended abdomen with an indentable mass in the lower left quadrant. Blood tests revealed acute renal failure (urea 16.1 mmol/L, creatinine 131 umol/L) and microcytic anaemia (Hb 7.7 g/dL, MCV 66.8 fL). CRP was 55 mg/L, and WCC was 9.1 × 10^9^/L.

**Figure 1:**
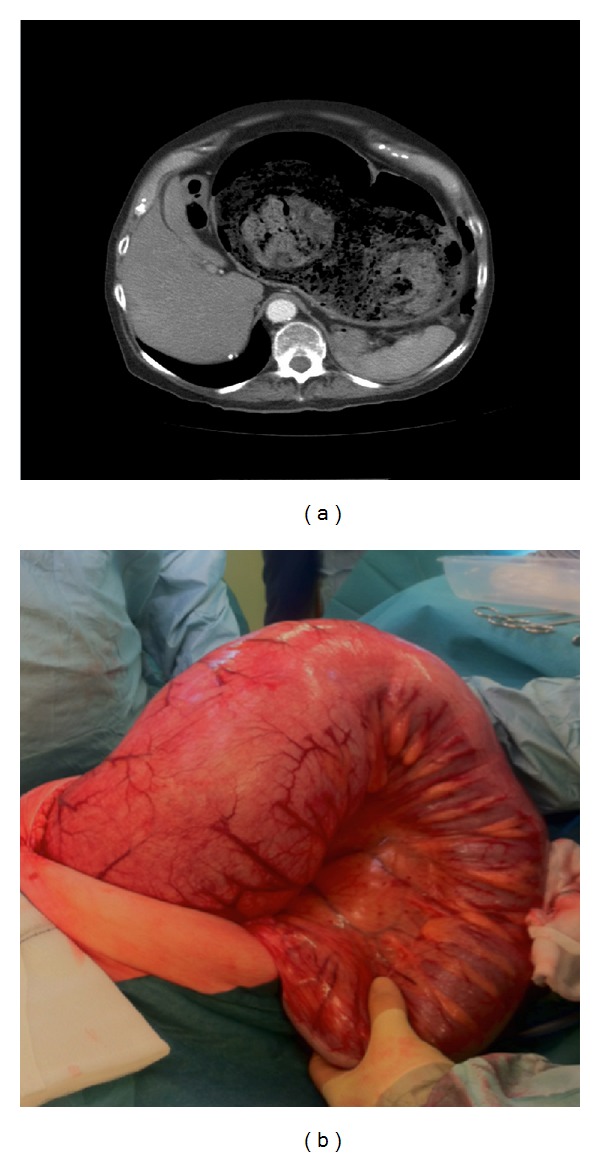
(a) CT scan showing grossly distended sigmoid containing faeces. (b) Sigmoid colon at laparotomy.

CT scan revealed massive dilatation of the sigmoid colon with faecal material within, measuring up to 17.9 cms in diameter, and no evidence of mechanical obstruction or perforation (Figure 1(a)), that is, no transition point was seen and the rectum was dilated and faecally loaded. She had resulting bilateral renal tract obstruction. Her abdominal tenderness and pain worsened, and a clinical decision was made to take her for laparotomy rather than endoscopy or contrast enema.

At laparotomy, she was found to have massive colonic dilatation of the sigmoid and descending colon (Figure 1(b)), but the transverse and right colons were only moderately dilated. There dense perirectal fibrosis throughout the length of the rectum. The rectum was resected at the mid rectum, and a Hartman's procedure was performed. Her renal function normalised, and she made an uneventful recovery and was discharged 10 days after the operation.

Histology from the resected specimen revealed that the longitudinal muscle layer was degenerate and absent in many areas of the specimen. The degenerate areas consisted largely of elastosis. The myenteric and submucosal plexuses were degenerate where the longitudinal muscle is absent. Accompanied with this, CD117 staining for the interstitial cells of Cajal was reduced. Also, polyglucosan inclusion bodies were present throughout the longitudinal and circular layers of the muscularis propria, with almost total degeneration of the longitudinal layer, suggesting a myopathic process. The occurrence of longitudinal loss was in areas where the overlying muscle and mucosa were intact indicating that this was likely to be a primary myopathy. There was also a replacement of myocytes in both muscularis layers with elastosis and fibrosis. Additional areas of elastosis were found in the submucosa, with widespread smooth muscle metaplasia, thought to be a secondary characteristic of disordered motility.

## 3. Discussion

This patient presented acutely with large bowel obstruction but had been suffering for many years from chronic constipation. Whilst it is rare for a patient to present in this way, chronic constipation in the elderly is a common and important problem. Understanding the aetiology may provide useful therapeutic strategies in the future [4].

Primary colonic myopathy should be considered as a potential diagnosis in elderly patients presenting with large bowel obstruction particularly when associated with a longstanding history of constipation as evidenced by this case. Endoscopic examination and biopsies may be unhelpful as the pathology lies in the muscularis layer of the colonic wall, and this diagnosis must be borne in mind when normal mucosal biopsies are seen.
